# Contact Experiences of Adolescents and Family Members Are Associated With Decrease of Personal Stigma But Increase of Perceived Stigma

**DOI:** 10.1002/jad.12519

**Published:** 2025-05-21

**Authors:** Sosei Yamaguchi, Shuntaro Ando, Atsushi Nishida, Kiyoto Kasai, Shinsuke Koike

**Affiliations:** ^1^ Department of Community Mental Health & Law, National Institute of Mental Health National Center of Neurology and Psychiatry Kodaira, Tokyo Japan; ^2^ Department of Neuropsychiatry, Graduate School of Medicine The University of Tokyo Bunkyo Tokyo Japan; ^3^ Department of Psychiatry and Behavioral Sciences Tokyo Metropolitan Institute of Medical Science Setagaya Tokyo Japan; ^4^ University of Tokyo Institute for Diversity and Adaptation of Human Mind, The University of Tokyo Meguro, Tokyo Japan; ^5^ The International Research Center for Neurointelligence (WPI‐IRCN) The University of Tokyo Institutes for Advanced Study (UTIAS) Bunkyo, Tokyo Japan; ^6^ Center for Evolutionary Cognitive Sciences, Graduate School of Arts and Sciences The University of Tokyo Meguro, Tokyo Japan

**Keywords:** adolescents, and personal stigma, behavioral intentions, contact experience, family, perceived stigma

## Abstract

**Background:**

Adolescents’ mental health‐related public stigma, encompassing personal and perceived stigma, may vary according to family dynamics and personal or familial experiences. This study aimed to investigate the association between adolescents’ and their family members’ stigma, specifically whether adolescents’ personal and perceived stigma are associated with other members’ experiences, particularly contact experiences.

**Methods:**

From a population‐based cohort of adolescents aged 17 years in Tokyo, 1,198 responses from 378 families (349 target adolescents, 364 mothers, 291 fathers, 194 siblings) were used in this study. Adolescents, their parents, and elder siblings responded to the self‐report questionnaires. Personal and perceived stigma were assessed using the behavioral intention subscale of the Reported and Intended Behavior Scale and Perceived Stigmatizing Attitude Scale, respectively. The relationship between stigma and contact experiences with people with mental health problems was examined simultaneously among adolescents and their family members.

**Results:**

The stigma of adolescents and their siblings was lower than that of their parents. Personal stigma of adolescents is associated with that of their siblings. Overall, contact experiences with people with mental health problems were associated with reduced personal and increased perceived stigma. Mothers’ contact experiences were associated with adolescents’ personal stigma.

**Conclusion:**

The stigma toward people with mental health problems may be shared between adolescents and siblings. Mothers’ contact experiences with people with mental health problems may be related to adolescents’ stigma. Family members’ stigma toward people with mental health problems and their contact experiences may play an important role in stigma formation among adolescents.

**Abbreviations**
CFI: comparative fit index
GLMs: general linear models
GLMMs: general linear mixed models
IQ: intelligence quotient
MHE‐9: 9‐item mental health‐related experience scale
PSAS: perceived stigmatizing attitude scale
RIBS‐J: Japanese version of the Reported and Intended Behavior Scale
RIBS‐J‐IB: Japanese version of the Reported and Intended Behavior Scale, intended behavior subscale
RMSEA: root mean square error of approximation
SEM: structural equation model
SES: socioeconomic status
SRMR: standardized root mean square

## 1 Introduction

Stigma toward people with mental health problems is a pervasive global issue. The general public often develops stigmatizing attitudes towards people with mental health problems during adolescence (Kaushik et al. 2016), which is a critical period for the onset of many mental disorders (Solmi et al. 2022). Adolescents’ attitudes are typically associated with their family environments. However, the extent to which adolescents’ and family's public stigma are related remains largely unknown. In addition, although previous relevant studies have emphasized the importance of contact experiences in reducing public stigma (Thornicroft et al. 2022), little is known about which family members’ contact experiences and other relevant experiences are associated with adolescents’ public stigma.

Public stigma encapsulates two facets: personal stigma and perceived stigma (Griffiths et al. 2004; Latalova et al. 2014). Personal stigma refers to an individual's own attitudes and responses towards people with mental health problems (Latalova et al. 2014) and is often assessed through questions about one's personal views (e.g., ‘I believe…’). Perceived stigma is defined as an individual's belief that others hold negative attitudes or stereotypes (Busby Grant et al. 2016; Latalova et al. 2014) typically measured by questions about others’ perceived thoughts (e.g., ‘Many people believe…’). Unlike personal stigma, which is one's own attitude to mental health problems, perceived stigma focuses on an individual's perception of broader society's or the general public's views of mental health problems. Among adolescents, ethnic and racial research indicates that ethnic minority groups who perceive lower public regard tend to view their own group as being of lower value and consequently exhibit heightened awareness of surrounding stigma (Gillen‐O'Neel et al. 2011; Johnson et al. 2005). Accordingly, personal and perceived stigma may be particularly interrelated during adolescence. Indeed, both personal and perceived stigma are generally associated with own help‐seeking behaviors and exclusive attitude. (Best and Bowie 2022; Clement et al. 2015; Morgan et al. 2007; Moses 2014; Schnyder et al. 2017; Villatoro et al. 2022; Woodgate et al. 2020). Especially, perceived stigma is strongly related to self‐stigma, leading to hesitation in seeking treatment and services or introducing services to others when needed (Yu et al. 2023). Given the peak age for the onset of mental disorders, investigating both personal and perceived stigma in the adolescent population appears to be particularly important.

The attitudes and thoughts of family members are a factor that contributes to public stigma. Adolescence represents a period of formation of attitudes towards others based on parents’ or siblings’ attitudes (Brody et al. 1994; McHale et al. 2012). Although individuals primarily acquire socialization and interpersonal skills by responding to parental behaviors during childhood (Grusec 2011; Sperling and Repetti 2018), parents continue to play a significant role in shaping their children's emotional development throughout adolescence (Miller‐Slough and Dunsmore 2016, 2020). As they transition into adolescence and spend less time with parents, peer groups—including siblings of similar age—exert increasing influence on their development trajectories (Smetana et al. 2015). In particular, younger siblings frequently model or adopt behavioral patterns exhibited by older siblings (Wang et al. 2019). These familial roles in adolescent development may also be related to the formation of public stigma.

Previous research has demonstrated the potential relationship between parents’ stigmatizing attitudes and their recognition of children's mental health problems, as well as the utilization of mental health services (Chandra and Minkovitz 2006; Gronholm et al. 2015; Villatoro et al. 2018). We also found a significant association between adolescent children's and mothers’ attitudes towards people with mental health problems (Koike et al. 2017). In addition, we previously reported the effect of repeated contact experiences on reducing personal stigma in young people for 2 years (Koike et al. 2018; Yamaguchi et al. 2019), and the effect was larger when mothers held greater personal stigma (Ojio et al. 2020). Moreover, adolescents are typically susceptible to the influence of others’ opinions, including stigmatizing attitudes, particularly those of same‐generation peers (Kaushik et al. 2016; Solomon and Knafo 2007). Siblings occupy a unique position as family members who typically share greater age proximity to adolescents than parents, yet maintain a distinct relationship that differentiates them from peers. Given that siblings frequently serve as models for adolescents’ development patterns (McHale et al. 2012), their stigmatizing attitudes may be associated with adolescents’ own attitudes. In other words, adolescents’ public stigma may be shaped by family relationships and the individual and shared experiences of the family members.

Apart from the family influences on stigma, a large body of literature has examined factors associated with personal stigma in adolescence. Systematic reviews have consistently underscored the positive impact of anti‐stigma interventions involving intended contact experience and education on reducing personal stigma (Goodwin et al. 2021; Janoušková et al. 2017; Morgan et al. 2018; Yamaguchi et al. 2013), while the effect sizes tend to be smaller in adolescents compared to adults (Corrigan et al. 2012). Additionally, some studies have reported adverse effects of such interventions (Ma et al. 2023; O'Mara et al. 2013). Moreover, both mainstream and social media—which adolescents frequently engage with—have been identified as potential shapers of personal stigma (Goodwin et al. 2016; Ross et al. 2019; Thornicroft et al. 2022). Thus, the effects of mental health‐related experiences may differ by age group. In addition, given the considerable influence of family members on adolescents’ socialization, each family member's mental health‐related experiences may be associated with the adolescents’ own personal stigma–related beliefs.

With regard to perceived stigma, the studies also examined the relevant factors. As opposite to personal stigma, a systematic review has failed to demonstrate a definitive effect of contact experience and education on diminishing perceived stigma (Griffiths et al. 2014). Observational studies have reported heterogeneous results on the associations between perceived stigma and contact experiences (Busby Grant et al. 2016; Manago and Krendl 2023). In particular, stigmatizing comments on social media have increased perceived stigma in young people (Dempsey et al. 2022). Thus, contact experience and other exposures to mental health information may be related to personal and perceived stigma differently. Considering adolescents’ development process, their perceived stigma may be connected to family members’ experiences, yet the available evidence remains limited.

Despite an extensive body of literature on public stigma in adolescents, few studies have simultaneously investigated personal stigma, perceived stigma, contact experience, and other exposures to mental health information within the family. To address this gap, this study aimed to analyze the associations between the level of personal and perceived stigma among adolescents and their family members, including mothers, fathers, and siblings. This study specifically focused on identifying the relationships between contact experience and stigmatizing attitudes of each family member group, namely target adolescents, mothers, fathers, and siblings, and whose (among family members) contact experiences were significantly associated with the levels of personal and perceived stigma in adolescents. To explore these relationships, we tested the following questions.

1. Do personal and perceived stigma differ among family members?
2. Which family members’ stigma correlates with adolescents’ personal and perceived stigma?
3. Are one's own contact experience, associated with personal and perceived stigma in adolescents, elder siblings, mothers, and fathers?
4. Are adolescents’ personal and perceived stigma associated with other family members’ contact experience?

## 2 Methods and Materials

### 2.1 Overall Design

The present research was a cross‐sectional study nested within a population‐based cohort study project targeting adolescent development in Japan (population‐neuroscience Tokyo TEEN Cohort: pn‐TTC) (Okada et al. 2019) and used an additional self‐reported questionnaire for mental health‐related stigma. The survey was conducted in 2019 and 2021. This study included adolescents aged 17 years and their family members (Table 1). The data were analyzed using a cross‐sectional design.

**Table 1:** Characteristics and scale scores of each attribution group.

	cohort members^a^		Mother		Father		Sibling		*p*	Difference
	(*n* = 349)		(*n* = 364)		(*n* = 291)		*n* = 194^b^			
**Sex**, *n* (%)										
**Female**	164	(47.0)	364	(100.0)	0	(0.0)	90	(46.4)	< 0.001	father < cohort member, siblings < mother
**Age at survey**, mean (SD)	16.6	(0.9)	49.4	(4.1)	51.6	(5.7)	19.5	(2.8)	< 0.001	cohort member < sibling < mother < father
**Stigmatizing attitudes scale**										
**Personal stigma [RIBS‐J‐IB]**, mean (SD)	13.6	(3.2)	12.6	(2.9)	12.00	(3.2)	13.4	(3.1)	< 0.001	cohort member, sibling > mother > father
**Perceived stigma [PSAS]**, mean (SD)	12.6	(2.7)	11.8	(2.7)	11.5	(2.7)	12.5	(2.9)	< 0.001	cohort member, sibling > mother, father
**MHE‐9**, Latent factor scores										
**Self‐experience**, mean (SD)	−0.16	(0.8)	0.13	(1.0)	0.05	(1.0)	−0.01	(0.9)	< 0.001	cohort member < mother, father, sibling
**Contact experience**, mean (SD)	−0.43	(0.5)	0.27	(0.9)	0.33	(0.9)	−0.21	(0.7)	< 0.001	cohort member < sibling < mother, father
**Lecture experience**, mean (SD)	−0.29	(0.8)	0.07	(1.0)	0.28	(1.1)	0.03	(1.0)	< 0.001	cohort member < sibling, mother < father

^a^ Adolescents of cohort members.

^b^ Two or more siblings in a family were responded from 19 families.

MHE‐9, 9‐item mental health‐related experience scale; PSAS, perceived stigmatizing attitude scale; RIBS‐J‐IB: Japanese version of the Reported and Intended Behavior Scale ‐ intended behavior subscale.

### 2.2 Participants

A total of 480 adolescent participants from the cohort sample and their family members were targeted for this survey. Of these 479 families, 409 were actively contacted during the baseline survey period. We sent an envelope that contained the questionnaires to the adolescents of cohort members, their mothers, fathers, and siblings aged 15 years or older. If there were two or more eligible siblings in a family, family members requested additional questionnaires for the siblings (Supporting Figure S1). All participants anonymously responded to the questionnaires, including the three scales for personal stigma, perceived stigma, and mental health‐related experiences, and posted themselves after completing the responses. This study was approved by the Ethics Committee of the Faculty of Medicine, The University of Tokyo (Approval No. 10069). The participants provided written informed consent before they responded to the questionnaire. For the adolescents and their siblings aged less than 18 years, the written informed consent from their parents was also provided.

We received 1,220 responses from 378 families. After the initial check, a total of 22 responses were excluded due to either incomplete data rendering one or more stigma scales unusable or inappropriate response patterns (e.g., the majority of items being rated as “1”). Finally, 1,198 valid responses from 378 families (349 adolescents of cohort members [age: mean ± SD = 16.6 ± 0.9, female = 164 (47.0%)], 364 mothers [age: 49.4 ± 4.1], 291 fathers [age: 51.6 ± 5.7], 194 sibling [age: 19.5 ± 2.8, female = 90 (46.4%)]) were used in this study (Table 1).

### 2.3 Variable and Questionnaire

#### 2.3.1 Demographic Variables

The key variables of family (target adolescents’ mother, family, and siblings) were identified through the questionnaire. IQ at age 10 of pn‐TTC members was estimated by a short version of the Wechsler Intelligence Scale for Children (WISC‐III), which consists of two subsets (Information and Picture Completion) (Inada and Kamio 2010). Familial SES at age 10 of pn‐TTC members was assessed using a parental questionnaire for the higher educational attainment of parents in the following scoring: 1. Primary school or lower, 2. Junior high school, 3. High school (leaving before graduation), 4. High school (graduated), 5. Vocational school, 2‐year college, or 4‐year college or university (leaving before graduation), 6. 4‐year college or university (graduation), 7. 6‐year university, postgraduate education, or higher (Kanata et al. 2016; Okada et al. 2014). Those who were analyzed in this study (n = 378) had a higher intelligence quotient (IQ) of the adolescents and familial socioeconomic status (SES) at age 10 than those who were not (n = 101; IQ: 110.0 vs. 105.4, *p* = 0.002; SES: 4.82 vs. 4.59, *p* = 0.008; Supporting Table S1).

#### 2.3.2 Stigmatizing Attitudes Against Mental Health Problems

##### Personal Stigma

The Japanese version of the Reported and Intended Behavior Scale (RIBS‐J) was employed, which is widely used to assess personal stigma by asking participants’ behavioral intention and past and present experiences (see the next section) regarding mental health problems (Evans‐Lacko et al. 2011). The validity and reliability of the Japanese version were confirmed (Yamaguchi et al. 2014). Specifically, we used the 4‐item intended behavior subscale (RIBS‐J‐IB; e.g., “In the future, I would be willing to live with someone with a mental health problem.”, Supporting Table S2). The options were ‘agree strongly’, ‘agree slightly’, ‘neither agree nor disagree’, ‘disagree slightly’, ‘disagree strongly,’ and ‘ do not know. For each item, a score of 5 was allocated for strong agreement, while a score of 1 was assigned to strong disagreement (range: 4–20). The response option ‘don't know’, is assigned a score of 3. Higher scores indicate less personal stigma. Cronbach's alpha for the RIBS‐J‐IB subscale in all responses of this study was 0.81.

##### Perceived Stigma

We included the Perceived Stigmatizing Attitude Scale (PSAS). This scale contained four items from the RIBS‐J‐IB subscale and two additional items (Supporting Table S2). Drawing on the Link's perceived devaluation‐discrimination scale (Link 1987), each item in PSAS was phrased with the subject “Most people” rather than “I” (e.g., “ In the future, most people would be willing to live with someone with a mental health problem”). The options and scoring were the same as those in the RIBS‐J‐IB scale. Cronbach's alpha for the PSAS in all responses of this study was 0.89. Although the possible range of the PSAS scale score was 6–30, the score was divided by 1.5, to easily compare with the RIBS‐J‐IB scale score. A lower PSAS score indicates that respondents recognized negative attitudes towards people with mental health problems in the general public more strongly.

#### 2.3.3 Mental Health‐Related Experiences Including Contact Experience

We obtained a 9‐item mental health‐related experience scale (MHE‐9). Of these, 4 items were originally included in the reported behavior subscale of the RIBS‐J (e.g., “Are you currently living with, or have you ever lived with, someone with a mental health problem?”) We added five items on the experience of the self and from lectures and media (Supporting S3). The options were ‘never’, ‘once’, ‘twice or more’, and ‘unknown’, and were scored as 0, 1, 2, and 0, respectively (range: 0 –18). We further explored the subscales of experience with mental health problems. We first tested an exploration factor analysis using a randomly split half of the data and showed three‐factor solutions (Supporting Figure S2 and Table S3). The factors contained 3 items about self‐experience (e.g., “Do you currently have, or have you ever had, a mental health problem?”), 5 items about contact experience (e.g., “Do you currently have, or have you ever had, a close friend with a mental health problem?”), and 1 item about lecture experience (“Do you currently take, or have you ever taken, any class or lecture about a mental health problem?”). Confirmatory factor analysis using the remaining half of the samples showed a good fit (chi square = 72.9, df = 25, *p* < 0.001, comparative fit index [CFI] = 0.955, root mean square error of approximation [RMSEA] = 0.057, standardized root mean square [SRMR] = 0.044; Supporting Figure S3). Cronbach's alpha scores for the self and contact subscales in this data set were 0.85 and 0.65, respectively. Since 4 items of the contact experience subscale contained those of the reported behavior subscale of the RIBS‐J scale, the Pearson correlation coefficient between the contact experience subscale and reported behavior subscale was 0.93 (*p* < 0.001).

### 2.4 Statistical Analysis

First, we tested the difference in personal stigma (RIBS‐J‐IB scores) or perceived stigma (PSAS scores) between family members (i.e., adolescent cohort members, mothers, fathers, and siblings) using general linear mixed models (GLMMs) including family as a random intercept because the responses vary according to family and two or more siblings’ responses from a family. Statistical analyses were performed using ‘lmer’ package in R server version 4.2.2. and statistical significance was set at *p* < 0.05. For the correlation in stigmatizing attitude scale scores between family members, we tested Pearson's pair‐wise correlation coefficients and corrected multiple comparisons using Bonferroni's correction (*p* < 0.05/6 pairs = 0.0083). In the correlation analyses, we excluded the second or later responses from siblings per family (n = 21) to fit the correlation matrix.

Second, to investigate whether personal stigma or perceived stigma would be associated with those from other family members, we applied general linear models (GLMs), including RIBS‐J‐IB scores (personal stigma) or PSAS scores (perceived stigma) as dependent variables, the corresponding scores of other family members as independent variables in the above correlation analyses, and age, sex, and familial SES as covariates. As the response patterns varied in family and we needed to avoid a decrease in the degree of freedom, we added the stigma score of family members (i.e., adolescent cohort members, mothers, or fathers) to each model. Therefore, we applied Bonferroni correction (*p* < 0.05/3 = 0.017) to the models.

Third, to explore the relationship between stigmatizing attitude scales, we applied GLMMs, including RIBS‐J‐IB (personal stigma) or PSAS (perceived stigma) scores as dependent variables, the three experience subscales (standardized scores of contact experience, self‐experience and lecture experience) as independent variables, attribution in family, age, sex, and familial SES as covariates, and family as a random intercept. The Bonferroni correction (*p* < 0.05/3 = 0.017) was applied to the models. To test whether the relationship between the stigmatizing attitude scale scores would alter according to attribution in family, we also tested the GLMMs by adding the interaction of experience by attribution in family to the above models. To avoid multicollinearity, we used interaction terms for each experience subscale in each model.

Fourth, to assess whether personal stigma or perceived stigma would be associated with other family members’ contact experiences, we applied GLMs including RIBS‐J‐IB and PSAS scores as dependent variables, contact experiences of the self and other family members as independent variables, and age, sex, and familial SES as covariates. We also applied model constructions and Bonferroni correction (*p* < 0.05/3 = 0.017) to the models.

Finally, we applied a structural equation model (SEM) to the significant relationships for illustrating the overall relationship using ‘lavaan’ package in R. Age, sex, IQ, and SES of TTC members were used as covariates in the model. To simplify the model, we excluded the second or later responses from siblings per family like the correlation analysis above. To fit the model with missing responses, a full‐information maximum likelihood method was applied. A model with CFI > 0.95, RMSEA < 0.08, and SRMR < 0.05 was considered a good fit (Kline 2015; Yuan et al. 2016).

## 3 Results

### 3.1 Differences and Correlations Between Family Members

A GLMM showed that the cohort members of adolescents and their siblings had less personal stigma (higher RIBS‐J‐IB scores) compared to their mothers and fathers, and the mothers had higher scores compared to the fathers (all *p* < 0.001, Table 1). Adolescent members and their siblings also obtained less perceived stigma (higher PSAS scores) than their mothers and fathers (all *p* < 0.001 except for siblings vs. mothers *p* = 0.004). With regard to the MHE‐9, the mean score of the self‐experience subscale in adolescent members was lower than that of their mothers, fathers, and siblings (all *p* < 0.001, except for adolescent members vs. siblings, *p* = 0.041). Adolescent members had lower scores on the contact experience subscale compared to their mothers, fathers, and siblings (all *p* < 0.001 except for adolescent members vs. siblings *p* = 0.001), and siblings had lower scores compared to their mothers and fathers (all *p* < 0.001). For the lecture experience subscale, adolescent members had lower scores compared to their mothers, fathers, and siblings (all *p* < 0.001), and fathers had higher scores compared to their mothers and siblings (*p* < 0.001 and *p* = 0.0053).

**Figure 1:**
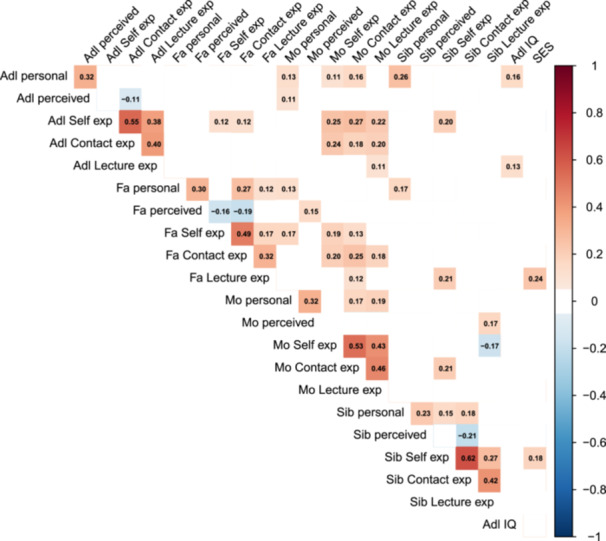
Correlation matrix of the stigmatizing attitudes scale and MHE‐9. The figure only exhibited correlations with small or large effect sizes (|*r* | > 0.1). The full correlation matrix is shown in the Supporting Figure S4. Adl, adolescents (cohort members); Contact, contact experience subscale scores of MHE‐9; Fa, Fathers; IQ, intelligence quotient; Mo, mothers; Lecture exp, lecture experience subscale score of MHE‐9; personal, (Japanese version of) Reported and Intended Behavior Scale, intended behavior subscale; perceived, Perceived Stigmatizing Attitude Scale; Self exp, self‐experience subscale scores of MHE‐9; SES, (familial) socioeconomic status; Sib, siblings.

A correlation matrix for the stigmatizing attitude scales, experience subscales of the MHE‐9, and demographic variables is shown in Fig. 1 for significant correlation coefficients and Supporting Figure 4 for all the results. A significant association was found between adolescent cohort members and siblings of their personal stigma (RIBS‐J‐IB score) (r = 0.26, *p* = 0.001) after Bonferroni correction, and a trend association between adolescent members and mothers of their personal stigma (r = 0.13, *p* = 0.018). For the experience subscales, significant associations were found between adolescent members and mothers of the self‐experience and contact experience subscales (r = 0.25, *p* < 0.001; r = 0.18, *p* = 0.001, respectively) and between fathers and mothers of the self‐experience and contact experience subscales (*r* = 0.19, *p* = 0.002; r = 0.25, *p* < 0.001, respectively).

### 3.2 Stigmatizing Attitude Scales Between Family Members

RIBS‐J‐IB scores for personal stigma in adolescent cohort members were associated with maternal scores (Model 2 in Supporting Table S4, B = 0.15, SE = 0.06, *t* = 2.42, *p* = 0.016) and sibling scores (Model 3, *B* = 0.26, SE = 0.08, *t* = 3.46, *p* < 0.001). The analysis (Model 4), which included all attributions, showed a significant association between the adolescent members’ and their siblings’ scores (B = 0.27, SE = 0.09, t = 3.07, *p* = 0.003). For the PSAS score for perceived stigma, there was no significant association between attributions.

### 3.3 Relationship Between Stigmatizing Attitude Scales and Experiences

**Figure 2:**
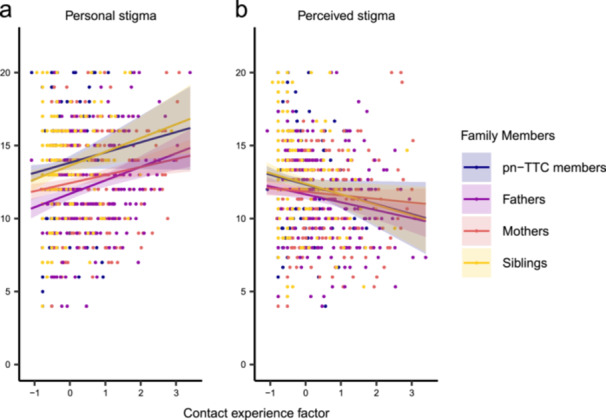
Relationship between contact experience and stigmatizing attitude scales. Personal and perceived stigmas were assessed using the Japanese version of Reported and Intended Behavior Scale, intended behavior subscale (RIBS‐J‐IB), and the Perceived Stigmatizing Attitude Scale (PSAS), respectively.

A GLMM including all the family members showed that contact experience factor scores were positively associated with RIBS‐J‐IB for personal stigma (*B* = 0.67, SE = 0.14, *t* = 4.76, *p* < 0.001; Figure 2a and Supporting Table S5) and negatively associated with PSAS scores for perceived stigma (*B* = −0.42, SE = 0.13, *t* = −3.27, *p* = 0.001; Figure 2b and Supporting Table S6). When adding experience X attribution interaction to the models, there was no significant interaction (*p* > 0.05), suggesting that the relationship between contact experiences and stigmatizing attitudes did not differ according to family members.

### 3.4 Relationship Between Stigmatizing Attitude and Contact Experiences of Other Family Members

Table 2 shows the associations between contact experiences and the stigmatizing attitude scale scores. When adding the corresponding contact experience subscale of another family member, personal stigma (RIBS‐J‐IB scores) of adolescent cohort members were associated with maternal contact experience (Model 3, *B* = 0.53, SE = 0.20, *t* = 2.63, *p* = 0.009) but not with self‐experience (*p* = 0.20). On the other hand, adolescent members’ contact experiences were significantly and negatively associated with their PSAS scores for perceived stigma (B = −0.77, SE = 0.34, *t* = −2.23, *p* = 0.026), while mothers’ contact experiences were not significantly related to adolescents’ perceived stigma scores (*p* = 0.410).

**Table 2:** Relationship of contact experience with adolescents’ (cohort members’) stigmatizing attitude scale scores.

	Model 1				Model 2				Model 3				Model 4			
Variable	B	SE	t	*p*	B	SE	t	*p*	B	SE	*t*	p	B	SE	t	p
Personal stigma [RIBS‐J‐IB]																
(Intercept)	**15.24**	**3.35**	**4.55**	**< 0.001**	**16.39**	**3.94**	**4.16**	**< 0.001**	**14.89**	**3.43**	**4.34**	**< 0.001**	**16.71**	**3.68**	**4.54**	**< 0.001**
Own^a)^ contact experience	0.72	0.38	1.88	0.061	**0.99**	**0.44**	**2.23**	**0.027**	0.51	0.39	1.29	0.200	0.33	0.65	0.50	0.620
Fathers’ contact experience					0.24	0.22	1.12	0.260								
Mothers’ contact experience									**0.53**	**0.20**	**2.63**	**0.009**				
Sibling's contact experience													0.30	0.45	0.67	0.500
Perceived stigma [PSAS]																
(Intercept)	**15.24**	**2.91**	**5.23**	**< 0.001**	**14.30**	**3.30**	**4.33**	**<0.001**	**14.54**	**3.02**	**4.81**	**< 0.001**	**14.34**	**3.31**	**4.32**	**< 0.001**
Own contact experience	−0.64	0.33	−1.95	0.052	−0.69	0.37	−1.86	0.063	**−0.77**	**0.34**	**−2.23**	**0.026**	**−1.16**	**0.58**	**−1.99**	**0.049**
Fathers’ contact experience					−0.09	0.18	−0.50	0.620								
Mothers’ contact experience									0.15	0.18	0.82	0.410				
Sibling's contact experience													‐0.66	0.41	−1.62	

a) Adolescents of cohort members.

Bold shows significant coefficients (*p* < 0.05).

All models included age, sex, and familial SES as covariates; however, these covariates were not significantly related in any model.

PSAS, perceived stigmatizing attitude scale; RIBS‐J‐IB: Japanese version of the Reported and Intended Behavior Scale ‐ intended behavior subscale.

**Figure 3:**
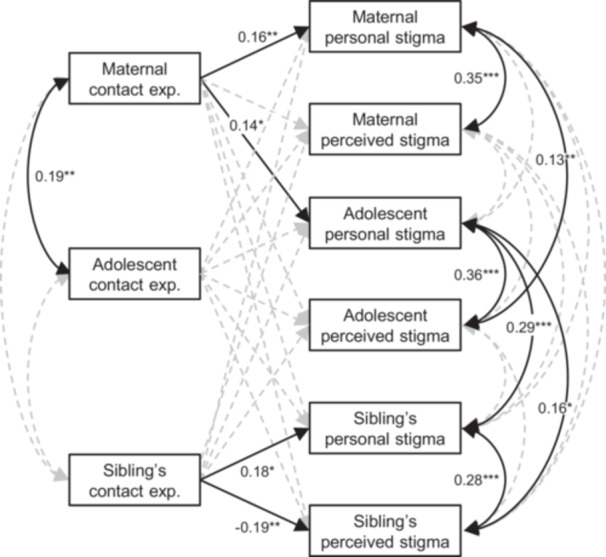
Association between contact experience and stigmatizing attitude scales in adolescent children, siblings and mothers. Adolescents, cohort members.

An SEM model including the personal stigma (RIBS‐J‐IB) and perceived stigma (PSAS) scores and the contact experience subscale scores of the adolescent members, as well as their siblings and mothers, showed a good fit (CFI = 1.0, RMSEA = 0.002, SRMR = 0.013). The model showed that maternal contact subscale scores were significantly associated with adolescent and maternal personal stigma scores (β = 0.14, SE = 0.06, z = 2.34, *p* = 0.019; β = 0.16, SE = 0.06, z = 2.91, *p* = 0.004, respectively; Figure 3 and Supporting Table S7). Maternal personal stigma scores were significantly associated with adolescent perceived stigma scores (*β* = 0.13, SE = 0.05, *z* = 2.60, *p* < 0.001). In contrast, the adolescent contact experience factor score was no longer significantly associated with the personal stigma and perceived stigma scores. For siblings, their contact experience factor scores were associated with their personal stigma and perceived stigma scores (*β* = 0.18, SE = 0.08, z = 2.14, *p* = 0.032; *β* = −0.19, SE = 0.07, z = ‐2.73, *p* = 0.006, respectively). Among siblings and adolescents, adolescent personal stigma scores were significantly associated with siblings’ personal stigma scores (β = 0.29, SE = 0.08, z = 3.59, *p* < 0.001) and perceived stigma scores (β = 0.16, SE = 0.08, z = 2.16, *p* = 0.031).

## 4 Discussion

This study examined the levels of personal and perceived stigma among adolescents and their family members. The findings revealed that adolescents and siblings exhibited lower levels of personal stigma, as indicated by higher scores on the RIBS‐J‐IB, as well as lower levels of perceived stigma, as indicated by higher scores on the PSAS scale, compared to their mothers and fathers. Overall, contact experiences were associated with reduced personal stigma, but higher perceived stigma when the analysis included all family members. Notably, both the regression models and the SEM model consistently indicated that adolescents’ personal stigma was significantly correlated with their siblings’ personal stigma and mothers’ contact experiences rather than their own experiences.

Regarding adolescents’ personal stigma, their siblings were the key family members to consider. Both adolescents’ and their siblings’ personal stigma scores were lower than those of their parents and were modestly correlated. Given their proximate ages, they are more likely to engage in frequent dialogs, including discussions related to mental health, compared to their parents (Smetana et al. 2015). In particular, older siblings frequently serve as role models for the socialization patterns of their younger siblings (Wang et al. 2019). These trends may be related to the low level of personal stigma held by adolescents in this study.

This study also identified contrasting overall trends, wherein contact experiences were associated with reduced personal stigma, but increased perceived stigma across all family members. Existing literature underscores the relationship between interpersonal contact experiences and positive personal attitudes toward people with mental health problems (Couture and Penn 2003; Maunder and White 2019; Pettigrew and Tropp 2006; Thornicroft et al. 2022). Structured and intended contact experience as part of anti‐stigma interventions is a well‐established strategy to mitigate both personal and perceived stigma (Maunder and White 2019; Thornicroft et al. 2022). However, a large‐scale study also suggested that the effect size of contact interventions on perceived stigma was less than that of personal stigma (Cerully et al. 2018). Additionally, spontaneous contact experiences in everyday life, such as those characterized by unfriendliness, unpleasantness, or very close relationships (e.g., roommates), have occasionally been linked to pronounced negative and problematic attitudes of rejecting other people with mental health problems (Eisenberg et al. 2012; Felix and Lynn 2022; Ran et al. 2022). Since the present study did not assess the qualitative nature of participants’ contact experiences and others’ reactions, some participants might witness others’ stigmatizing attitudes or discriminatory behaviors in their contact experiences, contributing to increased perceived stigma. The present findings suggest that mere contact experiences might amplify more perceived stigma even while being associated with less personal stigma.

The second finding from the multivariate analysis was the significant relationship between adolescents’ personal stigma and mothers’ contact experiences rather than adolescents’ own experiences. This finding aligns with broader sociological research indicating that parents, particularly mothers, exert considerable influence on various facets of their adolescent children's lives, including career choices, educational aspirations, and work values (Archer et al. 2014; Sassler et al. 2009; Schoon 2014). This influence may also extend to the mental health stigma domain. Furthermore, the moderate labor force participation rate for women in Japan (54%), as reported by the World Bank (2023), may suggest a larger maternal influence on adolescent children's developmental trajectories than paternal influence. Specifically, mothers’ prolonged engagement with their adolescent children implies increased opportunities for communication, and consequently, a higher likelihood that their experiences could shape their children's attitudes. Therefore, maternal contact experiences emerged as a potential factor associated with reduced personal stigma among adolescent children (adolescent members).

### 4.1 Strengths and Limitations

This study incorporated not only population‐based adolescents but also their siblings, mothers, and fathers, allowing for a comparative analysis of public stigma levels across the four groups of family members. The study also assessed both personal and perceived stigma (RIBS‐J‐IB and PSAS), which contain similar items, thereby facilitating a nuanced comparison between participants’ own future public stigma and their perceptions.

Concurrently, we recognize four major limitations of our study design. First, the cross‐sectional design of the study precludes the determination of causal relationships between variables. Second, although the study sought to explore the correlations of the stigma level between adolescents and their siblings, we could not include other same‐generation peer groups, such as classmates. Given that the nature and dynamics of adolescent relationships with peers and siblings could vary, a more expansive research scope that includes both these relational contexts is essential for a fuller understanding of the transmission mechanisms of public stigma. Third, the study employed newly developed instruments to assess perceived stigma and mental health‐related experiences. Although the factor structure of the MHE‐9 was verified, these tools may not have been sufficiently validated to reliably capture participants’ levels of perceived stigma and mental health experiences. Especially, lecture experience consists of only one item. Although there were few significant findings for the lecture experience in this study, the item should be carefully considered for further investigations. Fourth, representation bias could arise, as the analytic sample exhibited higher IQ and familial SES compared to those excluded from the study. In particular, recent research has reported that higher SES may correlate with stronger stigmatizing attitudes toward individuals experiencing mental health problems, potentially driven by competitive social worldviews (Foster 2021). Consequently, the relatively elevated SES of this sample may be associated with a higher level of public stigma than a broader population. Despite these limitations, this study enabled a direct comparison of public stigma levels between adolescents and their family members, as well as an examination of the relationship between adolescents’ public stigma and family members’ contact experiences. These findings may offer insights into anti‐stigma interventions that incorporate family involvement in adolescent populations.

## 5 Conclusion

This study investigated the association between personal and perceived stigma levels among adolescents and their family members. Adolescents’ and their siblings’ personal and perceived stigma were lower than that of their parents. Contact experiences were associated with less personal stigma and more perceived stigma across all four groups of family members. Adolescents’ personal stigma consistently showed significant associations with their siblings’ personal stigma and mothers’ contact experience rather than own contact experience. Future research should assess the qualitative nature of contact experiences and examine their relationship with various aspects of both personal and perceived stigma to provide more nuanced insights into the complexities of the stigma transmission process within the family.

## Author Contributions

Conceptualization: S.Y., S.A., and S.K.; Methodology: S.K.; Formal Analysis: S.K.; Investigation: S.K.; Resources: A.S. and S.K.; Data Curation: S.K.; Writing – Original Draft Preparation: S.Y. and S.K.; Writing – Review and Editing: S.Y., S.A., and S.K.; Visualization: S.Y. and S.K.; Project Administration: S.K.; Funding Acquisition: S.K. and S.A.

## Ethics Statement

The authors assert that all procedures contributing to this study comply with the ethical standards of the relevant national and institutional committees on human experimentation and the Helsinki Declaration of 1975, as revised in 2008. The ethical considerations of the current study, including the informed consent process and patient privacy measures, were based on the ethics guidelines for medical research in Japan. The study protocol was approved by the Research Ethics Committee of the Faculty of Medicine, University of Tokyo (approval no. 10069).

## Consent

The authors have nothing to report.

## Conflicts of Interest

The authors declare no conflicts of interest.

## Supporting information

The Supplemtary.

## Data Availability

The datasets generated and/or analyzed during the current study are available after approval by the ethics committee. Contact the corresponding author as requested.
