# Supplementary material for: Contact Experiences of Adolescents and Family Members Are Associated With Decrease of Personal Stigma But Increase of Perceived Stigma
Source: J Adolesc. 2025 May 21;97(6):1569–80. doi: 10.1002/jad.12519 (PMC12318468; doi:10.1002/jad.12519)
Supplement: Supplementary file 1 — The Supplemtary. [file JAD-97-1569-s001.docx]

**Online Supplementary Materials**

**Contents**

Supplementary Figure S1.

*Participant recruitment flow*  P.2

Supplementary Table S1.

*Demographic characteristics between those who participated in this study or not* P.2

Supplementary Table S2.

*Items in RIBS-J-IB for personal stigma and PSAS for perceived stigma* P.3

Supplementary Table S3.

*MHE-9 and the results of exploratory factor analysis* P.4

Supplementary Figure S2.

*Scree plot for the experience scale*  P.5

Supplementary Figure S3.

*Confirmatory factor analysis of the experience scale* P.5

Supplementary Figure S4.

*Full correlation matrix of the stigmatizing attitudes scale and MHE-9* P.6

Supplementary Table S4.

Relationship of adolescent stigma scales with those in other attributes

P7

Supplementary Table S5.

*Relationship of experience and attribution with personal stigma (RIBS-J-IB scale score)* P.8

Supplementary Table S6.

*Relationship of experience and attribution with perceived stigma (PSAS scale score)* P.9

Supplementary Table S7.

*Standardized estimation of the structural equation model*  P.10

**
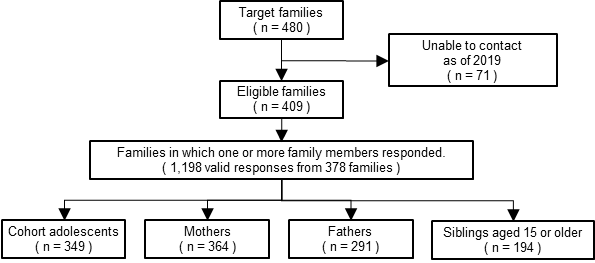
**

**Supplementary Figure S1. Participant recruitment flow**

**Supplementary Table S1.**

**Demographic characteristics between those who participated in this study or not**

|  | Participated  (n = 377) | Not participated  (N = 103) | P value |
| --- | --- | --- | --- |
| Sex, n (%) |  |  |  |
| Female | 174 (46.2) | 45 (43.7) | 0.740 |
| Intelligence quotient, mean (SD) | 110.0 (13.0) | 105.4 (12.6) | 0.002 |
| Familial SES, mean (SD) | 4.82 (0.76) | 4.59 (0.82) | 0.008 |

**Supplementary Table S2.**

**Items in RIBS-J-IB for personal stigma and PSAS for perceived stigma**

| Reported and Intended Behavior Scale, intended behavior subscale (RIBS-J-IB) |
| --- |
| 1. In the future, I would be willing to live with someone with a mental health problem. |
| 1. In the future, I would be willing to work with someone with a mental health problem. |
| 1. In the future, I would be willing to live near someone with a mental health problem. |
| 1. In the future, I would be willing to continue a relationship with a friend who developed a mental health problem. |
| **Perceived Stigmatizing Attitude Scale (PSAS)** |
| 1. Most people believe would be willing to trust someone with mental health problems as much as others. |
| 1. Most people would be willing to work with someone with a mental health problem. |
| 1. Most people would be willing to talk with someone with mental health problems as much as others. |
| 1. Most people could talk with someone with mental health problems as much as those without. |
| 1. Most people would be willing to live near someone with a mental health problem. |
| 1. Most people would be willing to live with someone with a mental health problem. |

The items 6, 2, 5, and 4 of the PSAS are corresponding to the RIBS-J-IB items 1, 2, 3, and 4, respectively. PSAS items 1 and 3 were made according to items 3 and 1 in the original Link’s stigma scale^1^ and item 3 in the Omnibus Survey in UK^2^.

1. Link BG: Understanding labeling effects in the area of mental disorders: An assessment of the effects of expectations of rejection. American Sociological Review 52(1):96-112, 1987. <https://doi.org/10.2307/2095395>
2. Crisp AH, Gelder MG, Rix S, Meltzer HI, Rowlands OJ: Stigmatisation of people with mental illnesses. British Journal of Psychiatry 177:4-7, 2000. <https://doi.org/10.1192/bjp.177.1.4>

**Supplementary Table S3.**

**MHE-9 and the results of exploratory factor analysis**

| No | Items | F1 | F2 | F3 |
| --- | --- | --- | --- | --- |
| V1 | **Have you ever taken, any class or lecture about a mental health problem?** | 0.01 | 0.00 | **0.99** |
| V2 | **Have you ever seen someone with mental health issues on television, in newspapers, or on the internet?** | 0.06 | 0.29 | 0.28 |
| V3 | **Do you currently have, or have you ever had, a mental health problem?** | **0.86** | 0.05 | 0.01 |
| V4 | **Have you ever discussed your mental health issues with family or close friends?"** | **0.79** | 0.09 | -0.03 |
| V5 | **Do you currently receive, or have you ever received treatment for mental health issues from a professional such as a doctor, psychologist, nurse, etc.?** | **0.81** | -0.11 | 0.05 |
| V6 | **Are you currently living with, or have you ever lived with, someone with a mental health problem?** | 0.17 | **0.43** | 0.00 |
| V7 | **Are you currently working with, or have you ever worked with, someone with a mental health problem?** | -0.14 | **0.52** | 0.16 |
| V8 | **Do you currently have, or have you ever had, a neighbor with a mental health problem?** | -0.02 | **0.57** | -0.07 |
| V9 | **Do you currently have, or have you ever had, a close friend with a mental health problem?** | 0.10 | **0.64** | 0.02 |
|  | With factor correlations |  |  |  |
|  | F1 |  | 0.31 | 0.38 |
|  | F2 |  |  | 0.38 |

Loadings of 0.30 or higher are bolded.

Abbreviations:

MHE-9, 9-item mental health-related experience scale

**
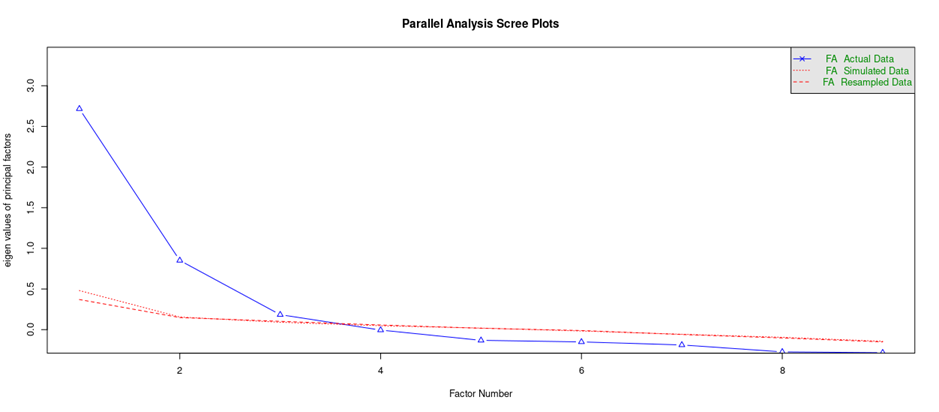
**

**Supplementary Figure S2. Scree plot for MHE-9**

Abbreviations: MHE-9, 9-item mental health-related experience scale


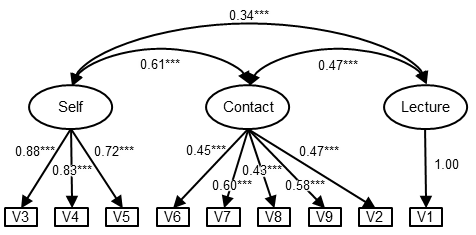


**Supplementary Figure S3. Confirmatory factor analysis of the MHE-9**

Abbreviations: MHE-9, 9-item mental health-related experience scale


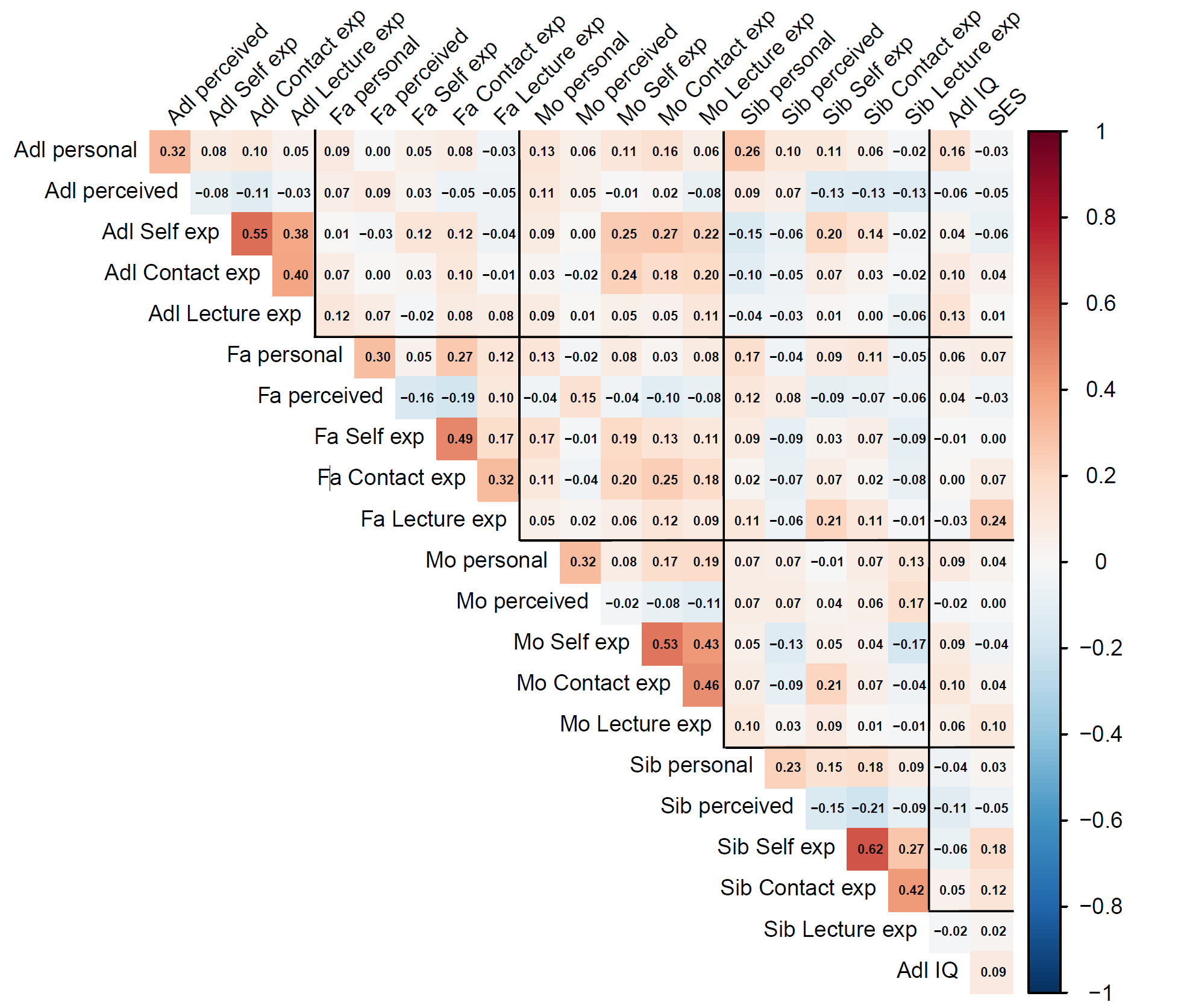


**Supplementary Figure S4.**

**Full correlation matrix of the stigmatizing attitudes scale and MHE-9**

Abbreviations (alphabetical order):

Adl, adolescents (cohort members); Contact exp, contact experience subscale scores of MHE-9; Fa, Fathers; IQ, intelligence quotient; Mo, mothers; Lecture exp, lecture experience subscale score of MHE-9; Perceived, perceived stigma measured by Perceived Stigmatizing Attitude Scale (PSAS); Personal, personal stigma measured by Japanese version of Reported and Intended Behavior Scale - intended behavior subscale (RIBS-J-IB); Self exp, self-experience subscale scores of MHE-9; SES, (familial) socioeconomic status; Sib, siblings

**Supplementary Table S4.**

**Relationship of adolescent stigma scales with those in other attributes**

|  | **Model 1** | | | | **Model 2** | | | | **Model 3** | | | | **Model 4** | | | |
| --- | --- | --- | --- | --- | --- | --- | --- | --- | --- | --- | --- | --- | --- | --- | --- | --- |
| Variable | **B** | **SE** | **t** | **p** | **B** | **SE** | **t** | **p** | **B** | **SE** | **t** | **p** | **B** | **SE** | **t** | **p** |
| Personal stigma  [RIBS-J-IB] |  |  |  |  |  |  |  |  |  |  |  |  |  |  |  |  |
| (Intercept) | **13.89** | **3.86** | **3.60** | **<0.001** | **12.72** | **3.33** | **3.82** | **<0.001** | **11.78** | **4.57** | **2.58** | **0.011** | 9.59 | 5.38 | 1.79 | 0.077 |
| Father | 0.10 | 0.06 | 1.62 | 0.11 |  |  |  |  |  |  |  |  | 0.13 | 0.08 | 1.55 | 0.13 |
| Mother |  |  |  |  | **0.15** | **0.06** | **2.42** | **0.016** |  |  |  |  | -0.03 | 0.10 | -0.28 | 0.78 |
| Sibling |  |  |  |  |  |  |  |  | **0.26** | **0.08** | **3.46** | **<0.001** | **0.27** | **0.09** | **3.07** | **0.003** |
| Perceived stigma  [PSAS] |  |  |  |  |  |  |  |  |  |  |  |  |  |  |  |  |
| (Intercept) | **14.70** | **3.28** | **4.48** | **<0.001** | **15.26** | **2.92** | **5.22** | **<0.001** | **16.60** | **4.29** | **3.87** | **<0.001** | **16.80** | **4.93** | **3.41** | **<.001** |
| Father | 0.10 | 0.06 | 1.55 | 0.12 |  |  |  |  |  |  |  |  | -0.00 | 0.09 | -0.02 | 0.98 |
| Mother |  |  |  |  | 0.05 | 0.05 | 0.97 | 0.34 |  |  |  |  | -0.07 | 0.09 | -0.83 | 0.41 |
| Sibling |  |  |  |  |  |  |  |  | 0.07 | 0.07 | 0.88 | 0.38 | 0.07 | 0.09 | 0.78 | 0.44 |

Bold shows significant coefficients (p < .05).

All the models included age, sex, and familial SES as covariates.

**Supplementary Table S5.**

**Relationship of experience and attribution with personal stigma (RIBS-J-IB scale score)**

|  | **Model 1** | | | | **Model 2** | | | | **Model 3** | | | | **Model 4** | | | |
| --- | --- | --- | --- | --- | --- | --- | --- | --- | --- | --- | --- | --- | --- | --- | --- | --- |
| Variable | **B** | **SE** | **t** | **p** | **B** | **SE** | **t** | **p** | **B** | **SE** | **t** | **p** | **B** | **SE** | **t** | **P** |
| (Intercept) | **13.69** | **0.77** | **17.75** | **<0.001** | **13.77** | **0.77** | **17.79** | **<0.001** | **13.74** | **0.79** | **17.48** | **<.001** | **13.65** | **0.77** | **17.64** | **<0.001** |
| Group |  |  |  |  |  |  |  |  |  |  |  |  |  |  |  |  |
| Mother | **-1.72** | **0.84** | **-2.06** | **0.039** | **-1.70** | **0.84** | **-2.03** | **0.042** | -1.61 | 0.85 | -1.90 | 0.057 | **-1.66** | **0.84** | **-1.97** | **0.049** |
| Father | **-2.41** | **0.89** | **-2.72** | **0.007** | **-2.42** | **0.89** | **-2.72** | **0.007** | **-2.43** | **0.90** | **-2.71** | **0.007** | **-2.34** | **0.89** | **-2.62** | **0.009** |
| Sibling | -0.31 | 0.27 | -1.15 | 0.251 | -0.34 | 0.27 | -1.24 | 0.217 | -0.28 | 0.32 | -0.88 | 0.381 | -0.26 | 0.28 | -0.93 | 0.355 |
| Experience |  |  |  |  |  |  |  |  |  |  |  |  |  |  |  |  |
| Self | -0.10 | 0.11 | -0.87 | 0.382 | 0.06 | 0.21 | 0.29 | 0.775 | -0.10 | 0.11 | -0.90 | 0.370 | -0.09 | 0.11 | -0.82 | 0.411 |
| Contact | **0.67** | **0.14** | **4.76** | **<0.001** | **0.69** | **0.14** | **4.89** | **<0.001** | 0.70 | 0.38 | 1.85 | 0.065 | **0.66** | **0.14** | **4.65** | **<0.001** |
| Lecture | 0.18 | 0.10 | 1.85 | 0.065 | 0.18 | 0.10 | 1.81 | 0.071 | 0.19 | 0.10 | 1.90 | 0.057 | 0.00 | 0.21 | 0.02 | 0.987 |
| Age | 0.01 | 0.02 | 0.35 | 0.724 | 0.01 | 0.02 | 0.31 | 0.756 | 0.01 | 0.02 | 0.28 | 0.777 | 0.01 | 0.02 | 0.34 | 0.737 |
| Sex | -0.02 | 0.23 | -0.08 | 0.936 | 0.00 | 0.23 | 0.02 | 0.987 | 0.00 | 0.23 | -0.02 | 0.983 | -0.02 | 0.23 | -0.08 | 0.936 |
| SES | 0.01 | 0.13 | 0.04 | 0.969 | 0.00 | 0.13 | -0.03 | 0.977 | 0.00 | 0.13 | 0.00 | 0.999 | 0.00 | 0.13 | 0.02 | 0.983 |
| Interaction |  |  |  |  |  |  |  |  |  |  |  |  |  |  |  |  |
| Self:Mo |  |  |  |  | -0.30 | 0.26 | -1.19 | 0.234 |  |  |  |  |  |  |  |  |
| Self:Fa |  |  |  |  | -0.33 | 0.27 | -1.22 | 0.222 |  |  |  |  |  |  |  |  |
| Self:Sib |  |  |  |  | 0.16 | 0.31 | 0.52 | 0.605 |  |  |  |  |  |  |  |  |
| Cont:Mo |  |  |  |  |  |  |  |  | -0.24 | 0.40 | -0.61 | 0.539 |  |  |  |  |
| Cont:Fa |  |  |  |  |  |  |  |  | 0.16 | 0.41 | 0.39 | 0.700 |  |  |  |  |
| Cont:Sib |  |  |  |  |  |  |  |  | 0.15 | 0.47 | 0.32 | 0.749 |  |  |  |  |
| Lec:Mo |  |  |  |  |  |  |  |  |  |  |  |  | 0.25 | 0.26 | 0.95 | 0.342 |
| Lec:Fa |  |  |  |  |  |  |  |  |  |  |  |  | 0.18 | 0.27 | 0.68 | 0.495 |
| Lec:Sib |  |  |  |  |  |  |  |  |  |  |  |  | 0.24 | 0.30 | 0.82 | 0.413 |

Bold shows coefficients of p < 0.05.

Abbreviations (alphabetical order):

Contact, contact experience; Fa, fathers; Lecture, lecture experience; Mo, mothers; Self, self-experience; SES, (familial) socioeconomic status; Sib, siblings

**Supplementary Table S6.**

**Relationship of experience and attribution with perceived stigma (PSAS scale score)**

|  | **Model 1** | | | | **Model 2** | | | | **Model 3** | | | | **Model 4** | | | |
| --- | --- | --- | --- | --- | --- | --- | --- | --- | --- | --- | --- | --- | --- | --- | --- | --- |
| Variable | **B** | **SE** | **t** | **p** | **B** | **SE** | **t** | **p** | **B** | **SE** | **t** | **p** | **B** | **SE** | **t** | **P** |
| (Intercept) | **13.17** | **0.69** | **19.12** | **<0.001** | **13.11** | **0.69** | **18.98** | **<0.001** | **13.03** | **0.70** | **18.52** | **<0.001** | **13.18** | **0.69** | **19.13** | **<0.001** |
| Group |  |  |  |  |  |  |  |  |  |  |  |  |  |  |  |  |
| Mother | 0.13 | 0.76 | 0.17 | 0.869 | 0.08 | 0.76 | 0.11 | 0.913 | 0.10 | 0.77 | 0.13 | 0.894 | 0.09 | 0.76 | 0.11 | 0.909 |
| Father | -0.24 | 0.81 | -0.30 | 0.765 | -0.25 | 0.81 | -0.31 | 0.757 | -0.18 | 0.82 | -0.22 | 0.830 | -0.35 | 0.81 | -0.43 | 0.665 |
| Sibling | -0.01 | 0.25 | -0.03 | 0.977 | 0.00 | 0.25 | 0.01 | 0.994 | 0.03 | 0.29 | 0.12 | 0.905 | 0.02 | 0.26 | 0.08 | 0.934 |
| Experience |  |  |  |  |  |  |  |  |  |  |  |  |  |  |  |  |
| Self | -0.13 | 0.10 | -1.21 | 0.225 | -0.18 | 0.19 | -0.93 | 0.351 | -0.11 | 0.10 | -1.07 | 0.283 | -0.10 | 0.10 | -0.97 | 0.334 |
| Contact | **-0.42** | **0.13** | **-3.27** | **0.001** | **-0.42** | **0.13** | **-3.27** | **0.001** | -0.65 | 0.34 | -1.89 | 0.059 | **-0.42** | **0.13** | **-3.27** | **0.001** |
| Lecture | 0.12 | 0.09 | 1.30 | 0.194 | 0.10 | 0.09 | 1.13 | 0.257 | 0.12 | 0.09 | 1.29 | 0.199 | 0.00 | 0.19 | 0.01 | 0.992 |
| Age | -0.02 | 0.02 | -0.88 | 0.382 | -0.02 | 0.02 | -0.83 | 0.406 | -0.02 | 0.02 | -0.79 | 0.429 | -0.02 | 0.02 | -0.78 | 0.436 |
| Sex | 0.12 | 0.21 | 0.54 | 0.588 | 0.11 | 0.21 | 0.53 | 0.596 | 0.10 | 0.21 | 0.46 | 0.646 | 0.09 | 0.21 | 0.45 | 0.656 |
| SES | -0.09 | 0.12 | -0.79 | 0.430 | -0.09 | 0.12 | -0.73 | 0.465 | -0.09 | 0.12 | -0.75 | 0.457 | -0.11 | 0.12 | -0.90 | 0.371 |
| Interaction |  |  |  |  |  |  |  |  |  |  |  |  |  |  |  |  |
| Self:Mo |  |  |  |  | 0.26 | 0.23 | 1.11 | 0.267 |  |  |  |  |  |  |  |  |
| Self:Fa |  |  |  |  | -0.09 | 0.25 | -0.36 | 0.721 |  |  |  |  |  |  |  |  |
| Self:Sib |  |  |  |  | -0.05 | 0.28 | -0.18 | 0.861 |  |  |  |  |  |  |  |  |
| Cont:Mo |  |  |  |  |  |  |  |  | 0.41 | 0.36 | 1.12 | 0.262 |  |  |  |  |
| Cont:Fa |  |  |  |  |  |  |  |  | 0.15 | 0.37 | 0.39 | 0.697 |  |  |  |  |
| Cont:Sib |  |  |  |  |  |  |  |  | 0.01 | 0.43 | 0.02 | 0.984 |  |  |  |  |
| Lec:Mo |  |  |  |  |  |  |  |  |  |  |  |  | -0.05 | 0.24 | -0.21 | 0.832 |
| Lec:Fa |  |  |  |  |  |  |  |  |  |  |  |  | 0.38 | 0.24 | 1.55 | 0.121 |
| Lec:Sib |  |  |  |  |  |  |  |  |  |  |  |  | 0.06 | 0.27 | 0.22 | 0.830 |

Bold shows coefficients of p < .05.

Abbreviations (alphabetical order):

Cont, contact experience; Fa, fathers; Lec, lecture experience; Mo, mothers; Self, self-experience; SES, (familial) socioeconomic status; Sib, siblings

**Supplementary Table S7.**

**Standardized estimation of structural equation modeling.**

|  | Relationship |  |  | Standard estimates | S.E. | z | p value | 95% C.I.  (Lower/Upper) | |
| --- | --- | --- | --- | --- | --- | --- | --- | --- | --- |
| 1 | Adl personal | ~ | Adl Contact | 0.067 | 0.072 | 0.937 | 0.349 | -0.073 | 0.207 |
| **2** | **Adl personal** | **~** | **Mo Contact** | **0.135** | **0.058** | **2.339** | **0.019** | **0.022** | **0.248** |
| 3 | Adl personal | ~ | Sib Contact | 0.037 | 0.073 | 0.498 | 0.618 | -0.107 | 0.181 |
| 4 | Adl personal | ~ | age | 0.008 | 0.058 | 0.137 | 0.891 | -0.105 | 0.121 |
| 5 | Adl personal | ~ | sex | -0.033 | 0.055 | -0.605 | 0.545 | -0.140 | 0.074 |
| 6 | Adl personal | ~ | IQ | 0.137 | 0.051 | 2.680 | 0.007 | 0.037 | 0.238 |
| 7 | Adl personal | ~ | SES | -0.049 | 0.052 | -0.953 | 0.340 | -0.151 | 0.052 |
| 8 | Mo personal | ~ | Adl Contact | -0.018 | 0.053 | -0.351 | 0.726 | -0.122 | 0.085 |
| **9** | **Mo personal** | **~** | **Mo Contact** | **0.162** | **0.056** | **2.913** | **0.004** | **0.053** | **0.271** |
| 10 | Mo personal | ~ | Sib Contact | 0.055 | 0.07 | 0.785 | 0.433 | -0.083 | 0.193 |
| 11 | Mo personal | ~ | age | 0.053 | 0.051 | 1.049 | 0.294 | -0.046 | 0.153 |
| 12 | Mo personal | ~ | sex | 0.044 | 0.054 | 0.814 | 0.416 | -0.062 | 0.150 |
| 13 | Mo personal | ~ | IQ | 0.082 | 0.052 | 1.582 | 0.114 | -0.020 | 0.184 |
| 14 | Mo personal | ~ | SES | 0.014 | 0.05 | 0.283 | 0.777 | -0.084 | 0.112 |
| 15 | Sib personal | ~ | Adl Contact | -0.152 | 0.088 | -1.732 | 0.083 | -0.324 | 0.02 |
| 16 | Sib personal | ~ | Mo Contact | 0.083 | 0.079 | 1.049 | 0.294 | -0.072 | 0.238 |
| **17** | **Sib personal** | **~** | **Sib Contact** | **0.184** | **0.084** | **2.191** | **0.028** | **0.019** | **0.349** |
| 18 | Sib personal | ~ | age | 0.096 | 0.082 | 1.174 | 0.240 | -0.064 | 0.257 |
| 19 | Sib personal | ~ | sex | -0.048 | 0.076 | -0.637 | 0.524 | -0.196 | 0.100 |
| 20 | Sib personal | ~ | IQ | -0.025 | 0.075 | -0.333 | 0.739 | -0.172 | 0.122 |
| 21 | Sib personal | ~ | SES | 0.002 | 0.073 | 0.032 | 0.975 | -0.140 | 0.144 |
| 22 | Adl perceived | ~ | Adl Contact | -0.106 | 0.059 | -1.811 | 0.070 | -0.221 | 0.009 |
| 23 | Adl perceived | ~ | Mo Contact | 0.053 | 0.058 | 0.907 | 0.364 | -0.061 | 0.166 |
| 24 | Adl perceived | ~ | Sib Contact | -0.119 | 0.074 | -1.614 | 0.107 | -0.263 | 0.025 |
| 25 | Adl perceived | ~ | age | -0.041 | 0.056 | -0.717 | 0.473 | -0.151 | 0.070 |
| 26 | Adl perceived | ~ | sex | -0.017 | 0.054 | -0.316 | 0.752 | -0.124 | 0.089 |
| 27 | Adl perceived | ~ | IQ | -0.043 | 0.054 | -0.800 | 0.424 | -0.150 | 0.063 |
| 28 | Adl perceived | ~ | SES | -0.027 | 0.053 | -0.505 | 0.614 | -0.131 | 0.077 |
| 29 | Mo perceived | ~ | Adl Contact | -0.020 | 0.059 | -0.343 | 0.732 | -0.135 | 0.095 |
| 30 | Mo perceived | ~ | Mo Contact | -0.077 | 0.066 | -1.166 | 0.244 | -0.206 | 0.052 |
| 31 | Mo perceived | ~ | Sib Contact | 0.064 | 0.076 | 0.834 | 0.404 | -0.086 | 0.214 |
| 32 | Mo perceived | ~ | age | 0.071 | 0.058 | 1.234 | 0.217 | -0.042 | 0.185 |
| 33 | Mo perceived | ~ | sex | -0.028 | 0.057 | -0.501 | 0.617 | -0.14 | 0.083 |
| 34 | Mo perceived | ~ | IQ | -0.008 | 0.052 | -0.158 | 0.874 | -0.111 | 0.094 |
| 35 | Mo perceived | ~ | SES | -0.006 | 0.054 | -0.117 | 0.907 | -0.113 | 0.1 |
| 36 | Sib perceived | ~ | Adl Contact | -0.036 | 0.083 | -0.440 | 0.660 | -0.198 | 0.126 |
| 37 | Sib perceived | ~ | Mo Contact | -0.073 | 0.077 | -0.954 | 0.340 | -0.224 | 0.077 |
| **38** | **Sib perceived** | **~** | **Sib Contact** | **-0.193** | **0.072** | **-2.674** | **0.007** | **-0.335** | **-0.052** |
| 39 | Sib perceived | ~ | age | 0.062 | 0.075 | 0.825 | 0.409 | -0.085 | 0.209 |
| 40 | Sib perceived | ~ | sex | 0.111 | 0.075 | 1.470 | 0.142 | -0.037 | 0.258 |
| 41 | Sib perceived | ~ | IQ | -0.072 | 0.072 | -0.994 | 0.320 | -0.213 | 0.07 |
| 42 | Sib perceived | ~ | SES | -0.039 | 0.086 | -0.449 | 0.654 | -0.207 | 0.13 |
| **43** | **Adl personal** | **~~** | **Adl perceived** | **0.355** | **0.052** | **6.869** | **< 0.001** | **0.254** | **0.456** |
| 44 | Adl personal | ~~ | Mo personal | 0.106 | 0.056 | 1.878 | 0.060 | -0.005 | 0.217 |
| 45 | Adl personal | ~~ | Mo perceived | 0.069 | 0.061 | 1.132 | 0.257 | -0.05 | 0.188 |
| **46** | **Adl personal** | **~~** | **Sib personal** | **0.294** | **0.082** | **3.586** | **< 0.001** | **0.133** | **0.455** |
| **47** | **Adl personal** | **~~** | **Sib perceived** | **0.164** | **0.076** | **2.158** | **0.031** | **0.015** | **0.314** |
| **48** | **Mo personal** | **~~** | **Adl perceived** | **0.130** | **0.050** | **2.596** | **0.009** | **0.032** | **0.229** |
| 49 | Adl perceived | ~~ | Mo perceived | 0.068 | 0.055 | 1.222 | 0.222 | -0.041 | 0.176 |
| 50 | Sib personal | ~~ | Adl perceived | 0.127 | 0.08 | 1.591 | 0.112 | -0.03 | 0.284 |
| 51 | Adl perceived | ~~ | Sib perceived | 0.075 | 0.091 | 0.82 | 0.412 | -0.104 | 0.254 |
| **52** | **Mo personal** | **~~** | **Mo perceived** | **0.349** | **0.053** | **6.64** | **< 0.001** | **0.246** | **0.452** |
| 53 | Mo personal | ~~ | Sib personal | 0.076 | 0.082 | 0.93 | 0.353 | -0.085 | 0.238 |
| 54 | Mo personal | ~~ | Sib perceived | 0.142 | 0.077 | 1.853 | 0.064 | -0.008 | 0.293 |
| 55 | Sib personal | ~~ | Mo perceived | 0.068 | 0.091 | 0.754 | 0.451 | -0.109 | 0.246 |
| 56 | Mo perceived | ~~ | Sib perceived | 0.105 | 0.07 | 1.497 | 0.134 | -0.032 | 0.242 |
| **57** | **Sib personal** | **~~** | **Sib perceived** | **0.282** | **0.077** | **3.675** | **< 0.001** | **0.131** | **0.432** |
| 58 | Adl Contact | ~ | age | 0.12 | 0.052 | 2.279 | 0.023 | 0.017 | 0.222 |
| 59 | Adl Contact | ~ | sex | -0.058 | 0.053 | -1.089 | 0.276 | -0.162 | 0.046 |
| 60 | Adl Contact | ~ | IQ | 0.099 | 0.053 | 1.883 | 0.060 | -0.004 | 0.202 |
| 61 | Adl Contact | ~ | SES | 0.03 | 0.062 | 0.482 | 0.630 | -0.091 | 0.151 |
| 62 | Mo Contact | ~ | age | -0.039 | 0.054 | -0.727 | 0.467 | -0.146 | 0.067 |
| 63 | Mo Contact | ~ | sex | 0.116 | 0.053 | 2.177 | 0.029 | 0.012 | 0.22 |
| 64 | Mo Contact | ~ | IQ | 0.096 | 0.055 | 1.765 | 0.078 | -0.011 | 0.203 |
| 65 | Mo Contact | ~ | SES | 0.018 | 0.057 | 0.306 | 0.759 | -0.095 | 0.13 |
| 66 | Sib Contact | ~ | age | -0.061 | 0.079 | -0.771 | 0.441 | -0.216 | 0.094 |
| 67 | Sib Contact | ~ | sex | 0.045 | 0.079 | 0.573 | 0.567 | -0.109 | 0.199 |
| 68 | Sib Contact | ~ | IQ | 0.049 | 0.063 | 0.784 | 0.433 | -0.074 | 0.172 |
| 69 | Sib Contact | ~ | SES | 0.108 | 0.069 | 1.556 | 0.120 | -0.028 | 0.244 |
| **70** | **Adl Contact** | **~~** | **Mo Contact** | **0.186** | **0.058** | **3.223** | **0.001** | **0.073** | **0.299** |
| 71 | Adl Contact | ~~ | Sib Contact | 0.044 | 0.093 | 0.477 | 0.633 | -0.137 | 0.226 |
| 72 | Mo Contact | ~~ | Sib Contact | 0.042 | 0.09 | 0.47 | 0.639 | -0.134 | 0.218 |
| 73 | age | ~~ | sex | 0 | 0 | NA | NA | 0 | 0 |
| 74 | age | ~~ | IQ | 0 | 0 | NA | NA | 0 | 0 |
| 75 | age | ~~ | SES | 0 | 0 | NA | NA | 0 | 0 |
| 76 | sex | ~~ | IQ | 0 | 0 | NA | NA | 0 | 0 |
| 77 | sex | ~~ | SES | 0 | 0 | NA | NA | 0 | 0 |
| 78 | IQ | ~~ | SES | 0 | 0 | NA | NA | 0 | 0 |
| 79 | Adl personal | ~~ | Adl personal | 0.945 | 0.026 | 36.684 | < 0.001 | 0.895 | 0.996 |
| 80 | Mo personal | ~~ | Mo personal | 0.955 | 0.023 | 42.052 | < 0.001 | 0.911 | 1.000 |
| 81 | Sib personal | ~~ | Sib personal | 0.938 | 0.039 | 24.205 | < 0.001 | 0.862 | 1.014 |
| 82 | Adl perceived | ~~ | Adl perceived | 0.967 | 0.023 | 41.31 | < 0.001 | 0.921 | 1.013 |
| 83 | Mo perceived | ~~ | Mo perceived | 0.984 | 0.016 | 59.71 | < 0.001 | 0.952 | 1.016 |
| 84 | Sib perceived | ~~ | Sib perceived | 0.928 | 0.038 | 24.628 | < 0.001 | 0.854 | 1.001 |
| 85 | Adl Contact | ~~ | Adl Contact | 0.972 | 0.018 | 53.671 | < 0.001 | 0.936 | 1.007 |
| 86 | Mo Contact | ~~ | Mo Contact | 0.975 | 0.015 | 63.067 | < 0.001 | 0.945 | 1.006 |
| 87 | Sib Contact | ~~ | Sib Contact | 0.98 | 0.021 | 46.651 | < 0.001 | 0.939 | 1.021 |
| 88 | age | ~~ | age | 1 | 0 | NA | NA | 1 | 1 |
| 89 | sex | ~~ | sex | 1 | 0 | NA | NA | 1 | 1 |
| 90 | IQ | ~~ | IQ | 1 | 0 | NA | NA | 1 | 1 |
| 91 | SES | ~~ | SES | 1 | 0 | NA | NA | 1 | 1 |
| 92 | Adl personal | ~ | 1 (Intercept) | 3.405 | 1.257 | 2.709 | 0.007 | 0.941 | 5.868 |
| 93 | Mo personal | ~ | 1 (Intercept) | 2.446 | 1.119 | 2.186 | 0.029 | 0.253 | 4.638 |
| 94 | Sib personal | ~ | 1 (Intercept) | 2.755 | 1.724 | 1.598 | 0.110 | -0.624 | 6.134 |
| 95 | Adl perceived | ~ | 1 (Intercept) | 5.809 | 1.204 | 4.826 | < 0.001 | 3.450 | 8.168 |
| 96 | Mo perceived | ~ | 1 (Intercept) | 3.241 | 1.275 | 2.543 | 0.011 | 0.743 | 5.740 |
| 97 | Sib perceived | ~ | 1 (Intercept) | 3.459 | 1.628 | 2.125 | 0.034 | 0.268 | 6.650 |
| 98 | Adl Contact | ~ | 1 (Intercept) | -3.960 | 1.158 | -3.420 | 0.001 | -6.230 | -1.691 |
| 99 | Mo Contact | ~ | 1 (Intercept) | -0.268 | 1.135 | -0.236 | 0.814 | -2.492 | 1.956 |
| 100 | Sib Contact | ~ | 1 (Intercept) | -0.572 | 1.672 | -0.342 | 0.732 | -3.849 | 2.706 |
| 101 | age | ~ | 1 (Intercept) | 18.212 | 0.599 | 30.382 | < 0.001 | 17.037 | 19.387 |
| 102 | sex | ~ | 1 (Intercept) | 3.069 | 0.063 | 48.406 | < 0.001 | 2.944 | 3.193 |
| 103 | IQ | ~ | 1 (Intercept) | 8.502 | 0.286 | 29.729 | < 0.001 | 7.941 | 9.062 |
| 104 | SES | ~ | 1 (Intercept) | 6.326 | 0.279 | 22.679 | < 0.001 | 5.779 | 6.872 |

Bold shows coefficients of p < .05 shown in Figure 3.

Abbreviations (alphabetical order):

Adl, adolescents (cohort members); Contact, contact experience subscale scores of MHE-9; IQ, intelligence quotient; Mo, mothers; Perceived, perceived stigma measured by Perceived Stigmatizing Attitude Scale (PSAS); Personal, personal stigma measured by Japanese version of Reported and Intended Behavior Scale - intended behavior subscale (RIBS-J-IB); SES, (familial) socioeconomic status; Sib, siblings
